# Functional trajectories beyond 90 days after endovascular thrombectomy: a systematic review with primary synthesis and supportive narrative evidence

**DOI:** 10.3389/fneur.2026.1847994

**Published:** 2026-08-03

**Authors:** Chenyang Zhao, Yi Han, Yaxuan Sun

**Affiliations:** Department of Neurology, Shanxi Provincial People’s Hospital, Shanxi Medical University, Taiyuan, China

**Keywords:** acute ischemic stroke, delayed functional improvement, endovascular thrombectomy, late functional deterioration, long-term outcome, modified Rankin Scale, systematic review

## Abstract

**Background:**

The modified Rankin Scale (mRS) score at 90 days is the conventional functional endpoint in studies of endovascular thrombectomy (EVT). However, it remains uncertain whether functional status after EVT is truly stable by 90 days. Although an increasing number of studies report outcomes at 1 year or later, only a small proportion directly examine within-patient functional change beyond the conventional 90-day time point.

**Methods:**

We performed a systematic review of functional trajectories beyond 90 days after EVT. PubMed, Embase, Web of Science, and the Cochrane Library were searched. Studies were screened using a hierarchical framework to distinguish directly eligible post-90-day trajectory studies from studies providing broader supportive evidence on long-term outcome. Because the directly eligible literature was limited and heterogeneous with respect to study population, follow-up structure, denominator definition, and transition operationalization, we conducted a structured primary synthesis rather than a conventional pooled meta-analysis.

**Results:**

A total of 283 records were identified, of which 52 full-text reports were assessed for eligibility. Fourteen studies were retained in the final review; however, only 3 studies directly examined within-patient functional transition beyond the conventional 90-day endpoint and were included in the primary synthesis, whereas 11 studies were retained as supportive or discussion-only evidence. The directly eligible studies showed that functional outcome after EVT is not invariably fixed at 90 days. Two studies identified delayed functional improvement beyond the conventional endpoint, whereas one multicenter study showed that late functional deterioration between 90 days and 1 year occurred in 21.5% of patients with vertebrobasilar artery occlusion. Taken together, the directly eligible evidence suggests that post-90-day functional outcome after EVT may be bidirectional, with both later recovery and later decline observed.

**Conclusion:**

Current evidence suggests that 90-day outcome after EVT may not always represent final recovery. Both delayed functional improvement and late functional deterioration appear possible beyond the conventional short-term endpoint, but the certainty of current evidence remains low. These findings should therefore be regarded as hypothesis-generating and support the need for prospective, standardized, trajectory-based follow-up after EVT.

## 1 Introduction

### 1.1 EVT established 90-day mRS as the conventional endpoint

Endovascular thrombectomy (EVT) has transformed the treatment of acute ischemic stroke caused by large-vessel occlusion. Landmark randomized trials established EVT as an effective reperfusion therapy and consistently demonstrated functional benefit in appropriately selected patients (1–4). Across these trials, outcome assessment at 90 days using the modified Rankin Scale (mRS) became the dominant measure of treatment effect, providing a common framework for comparing disability outcomes across studies and populations (1–4). This convention was subsequently reinforced by guideline recommendations and is now widely accepted as the standard endpoint for evaluating clinical efficacy after EVT (5).

The widespread adoption of the 90-day mRS reflects several practical and methodological strengths. It captures disability beyond the acute phase while remaining feasible for trial follow-up, and it provides a clinically interpretable measure of global functional status familiar to both stroke clinicians and researchers (1–5). Accordingly, 90-day outcome is now routinely used not only in randomized trials, but also in observational EVT studies, registry analyses, and clinical discussions of prognosis.

### 1.2 Endpoint convention does not necessarily equal biological finality

Despite its utility, a conventional endpoint should not be equated with a biologically final state. The widespread use of the 90-day mRS does not necessarily imply that recovery is complete by that time point. Neurological improvement may continue beyond 3 months through delayed biological recovery, rehabilitation-related gains, adaptation to disability, and restoration of function not fully captured during the early post-stroke period (5). Conversely, functional status may worsen after 90 days because of recurrent vascular events, medical complications, frailty, or an unstable recovery trajectory (5).

This distinction is clinically important. In routine practice, the 90-day endpoint is often interpreted as the time at which functional outcome after stroke has largely stabilized. However, this assumption may oversimplify the longer course of recovery after EVT, particularly in patients with severe initial deficits, posterior circulation stroke, or complicated post-acute recovery. Thus, although 90-day outcome remains highly useful, it may be better regarded as an important milestone rather than an automatic proxy for final recovery.

### 1.3 The true gap is not lack of long-term data, but lack of post-90-day transition analysis

The key unresolved issue is not simply whether long-term outcome data exist, but whether the literature directly captures within-patient functional transition beyond 90 days. A growing number of EVT studies report outcomes at 1 year or later; however, reporting a late endpoint is not equivalent to analyzing functional trajectory. A study may demonstrate a 1-year mRS distribution or sustained treatment effect without showing whether individual patients improved, remained stable, or deteriorated after the conventional 90-day assessment.

Only a limited number of studies have directly addressed this question. Todo et al. (6) suggested that successful reperfusion with EVT may confer benefit beyond the conventional endpoint. Gunasekera et al. (7) showed that functional recovery after basilar artery thrombectomy may continue beyond 3 months among long-term survivors. In contrast, Wang et al. (8) demonstrated that functional deterioration between 90 days and 1 year is also clinically relevant after EVT for vertebrobasilar artery occlusion. Taken together, these studies suggest that post-90-day outcome after EVT is not uniformly fixed and may instead be bidirectional.

### 1.4 Study objective

We therefore conducted a systematic review to examine functional trajectories beyond 90 days after EVT. Specifically, we sought to identify studies directly assessing within-patient functional change beyond the conventional 90-day endpoint, distinguish the available evidence on delayed functional improvement and late functional deterioration, and retain broader long-term EVT studies as supportive contextual evidence when they informed interpretation of post-90-day recovery. In the present review, “primary synthesis” refers specifically to the structured synthesis of studies directly examining post-90-day within-patient functional transition. Because the directly eligible evidence base is limited and conceptually distinct from the broader long-term EVT literature, the present review was designed as a systematic review with primary synthesis and supportive narrative evidence rather than as a conventional pooled meta-analysis (6–8).

## 2 Methods

### 2.1 Study design and reporting framework

This study was conducted as a systematic review of functional trajectories beyond the conventional 90-day endpoint after endovascular thrombectomy. The primary objective was to determine whether patients experienced delayed functional improvement after 90 days or late functional deterioration during longer-term follow-up. Reporting was structured with reference to the PRISMA 2020 statement (10). Because the directly eligible evidence base was small and heterogeneous in several clinically important domains, including study population, follow-up anchor, denominator structure, and transition definition, the review was designed as a structured primary synthesis with supportive narrative contextualization rather than as a conventional pooled meta-analysis. This approach was conceptually aligned with the SWiM framework for reviews in which formal meta-analysis is not appropriate or may be misleading (9).

### 2.2 Search strategy

A structured literature search was conducted in PubMed, Embase, Web of Science Core Collection, and the Cochrane Library from database inception to 14 April 2026. The search strategy combined terms related to ischemic stroke and vascular occlusion, endovascular thrombectomy, longer-term follow-up, and functional transition beyond the conventional 90-day endpoint. English-language and article-type records were retained in the final screened set where applicable. All retrieved records were exported into EndNote for reference management and duplicate handling. Full database-specific search strategies are provided in Supplementary Appendix 1.

### 2.3 Eligibility criteria

Studies were considered eligible if they included EVT-treated ischemic stroke populations and reported functional outcome beyond the conventional 90-day endpoint. During full-text review, studies were classified into three hierarchical groups to preserve the distinction between directly relevant trajectory evidence and broader long-term contextual evidence (9).

#### 2.3.1 Primary synthesis studies

Primary synthesis studies were defined as studies directly assessing within-patient functional transition beyond 90 days. Eligible studies in this category explicitly evaluated whether patients improved, remained stable, or deteriorated between the conventional 90-day assessment and a later follow-up point, or between 3 months and a later time point when the analysis clearly addressed post-conventional-endpoint change.

#### 2.3.2 Supportive/discussion-only evidence

Supportive or discussion-only evidence comprised studies reporting longer-term EVT outcomes without explicit analysis of within-patient post-90-day transition. These studies were retained only when they contributed contextual evidence relevant to the interpretation of long-term recovery, such as prognosis, durability of EVT benefit, etiologic or phenotypic heterogeneity, or outcomes associated with rescue strategies. Accordingly, these studies were considered clinically relevant to the broader topic of long-term EVT outcome, but not methodologically eligible for the primary synthesis.

#### 2.3.3 Exclusion criteria

Studies were excluded from the retained review set if they were non-trajectory studies without long-term interpretive value, prognostic-only studies lacking relevance to the broader recovery question, treatment-effect durability studies without contextual value for post-90-day interpretation, rescue-strategy or technique-specific studies not informative for long-term functional interpretation, review articles, guidelines, non-original reports, wrong-population studies, or reports otherwise not directly relevant to the present review question. This hierarchical screening framework was intended to avoid conflating long-term outcome reporting with direct trajectory analysis (9).

### 2.4 Study selection

**Figure 1:**
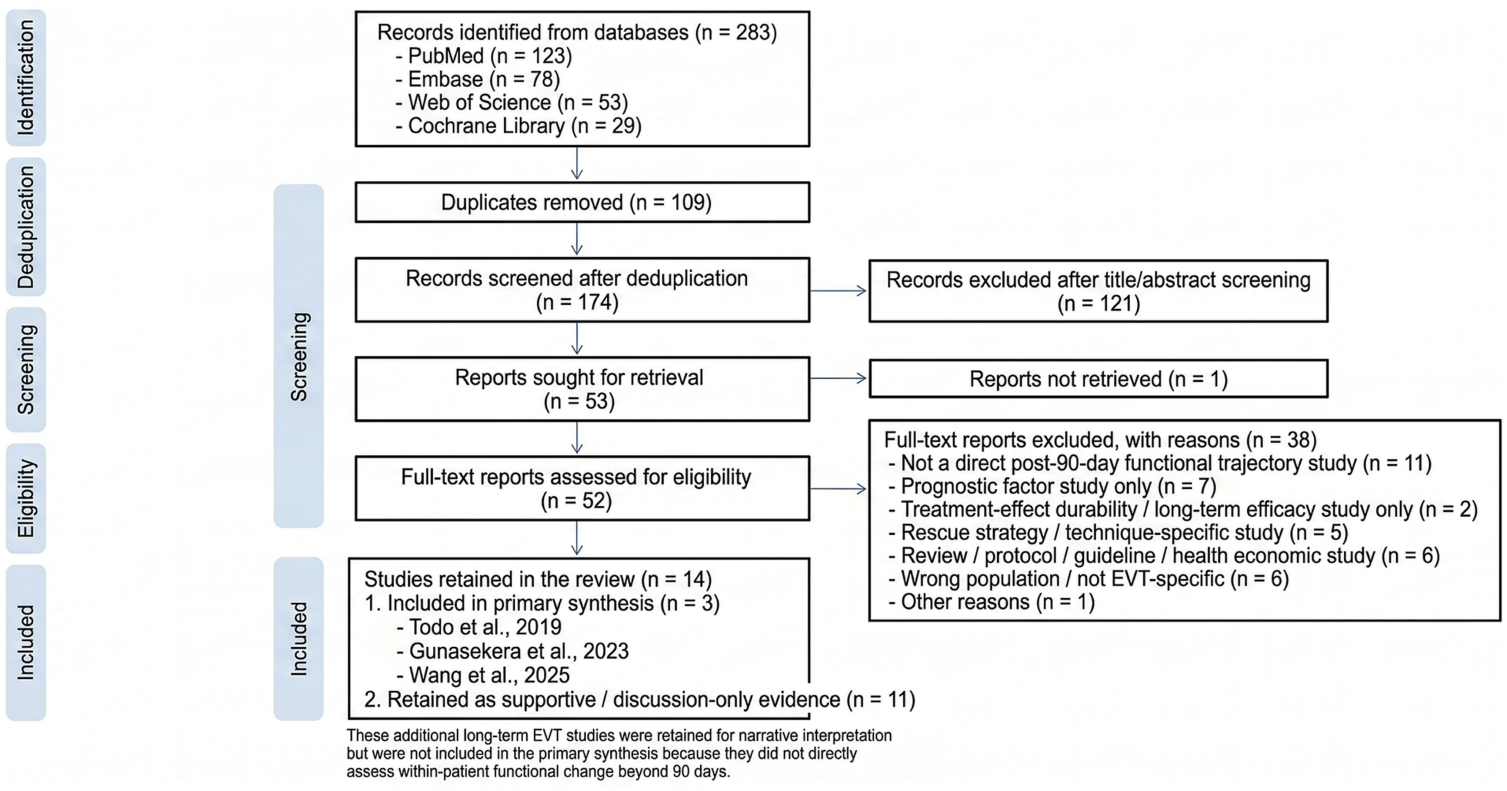
PRISMA flow diagram of study selection. A total of 283 records were identified from PubMed, Embase, Web of Science, and the Cochrane Library. After duplicate removal, 174 records underwent title/abstract screening, and 121 were excluded at this stage. Fifty-three reports were sought for retrieval, of which 1 could not be retrieved. Fifty-two full-text reports were assessed for eligibility, and 38 were excluded with specific reasons. Fourteen studies were retained in the final review, including 3 studies in the primary synthesis and 11 studies retained as supportive or discussion-only evidence. A full list of full-text reports excluded after eligibility assessment and their specific reasons for exclusion is provided in Supplementary Table S2.

A total of 283 records were identified through database searching. After reference management and duplicate removal, 174 records remained for title/abstract screening. Of these, 121 were excluded after title/abstract screening, and 53 reports were sought for retrieval. One report could not be retrieved, leaving 52 full-text reports assessed for eligibility. Of these, 38 were excluded and 14 were retained in the final review. Among the retained studies, 3 met criteria for the primary synthesis and 11 were retained as supportive or discussion-only evidence. The study selection process is summarized in Figure 1. Full-text reports excluded after eligibility assessment and their specific reasons for exclusion are listed in Supplementary Table S2.

### 2.5 Data extraction

Data extraction was performed in a structured manner. For studies included in the primary synthesis, extracted variables included study design, study population, occlusion territory, follow-up points, operational definition of post-90-day transition, trajectory category, and key quantitative findings. When available, numerical data on delayed improvement, late deterioration, stable status, and predictors of later worsening were also extracted.

For studies retained as supportive evidence, extraction focused on study type, principal long-term research focus, reason for exclusion from the primary synthesis, and interpretive role in the present review. This approach allowed the supportive literature to be summarized transparently without implying that it provided direct evidence on post-90-day functional transition. The resulting extraction framework underlies Tables 1, 2 and Supplementary Table S1.

**Table 1:** Characteristics of studies included in the primary synthesis.

Study	Country	Population	Territory	N	Follow-up	Trajectory domain	Key definition/role
Todo et al. (6)	Japan	EVT-treated AIS patients	LVO	NR	90 days, 1 year	Delayed functional improvement	90-day vs. 1-year functional change; core study directly addressing delayed recovery beyond 90 days.
Gunasekera et al. (7)	Australia	EVT-treated BAO patients	BAO	NR	3 months, 12 months	Delayed recovery in long-term survivors	3-month to 12-month functional reassessment; survivor selection should be acknowledged.
Wang et al. (8)	China	VBAO patients treated with EVT	VBAO	1,342	90 days, 1 year	Late functional deterioration	Deterioration defined as ≥ 1-point worsening in mRS from 90 days to 1 year; key complementary study showing late worsening after EVT.

EVT, endovascular thrombectomy; AIS, acute ischemic stroke; LVO, large-vessel occlusion; BAO, basilar artery occlusion; VBAO, vertebrobasilar artery occlusion; mRS, modified Rankin Scale; NR, not reported.

**Table 2:** Main findings of studies included in the primary synthesis.

Study	Follow-up	Concept	Key finding	Quantitative result	Interpretation
Todo et al. (6)	90 days, 1 year	Delayed functional improvement	A subset of EVT-treated patients continued to improve between 90 days and 1 year.	Delayed improvement: 46/268 (17.2%); deterioration: 84/268 (31.3%); stable: 138/268 (51.5%).	Core evidence supporting continued recovery beyond 90 days after EVT.
Gunasekera et al. (7)	3 months, 12 months	Delayed recovery in long-term survivors	Functional improvement beyond 3 months was observed in a subset of surviving BAO patients.	Improvement: 21/51 (41.2%); deterioration: 8/51 (15.7%); stable: 22/51 (43.1%).	Supportive evidence that recovery may continue beyond the traditional short-term endpoint.
Wang et al. (8)	90 days, 1 year	Late functional deterioration	Among 1,342 VBAO patients treated with EVT, 288 deteriorated between 90 days and 1 year.	Deterioration rate: 288/1,342 (21.5%); predictors included female sex, age, door-to-puncture time, and ≥ 4 thrombectomy passes.	Complementary evidence showing that late worsening, in addition to delayed recovery, occurs after EVT.

EVT, endovascular thrombectomy; BAO, basilar artery occlusion; VBAO, vertebrobasilar artery occlusion; OR, odds ratio.

### 2.6 Protocol and review process

This review was not prospectively registered. However, the review question, eligibility framework, and synthesis approach were prespecified before full-text synthesis.

### 2.7 Risk-of-bias assessment

Risk of bias was assessed only for studies included in the primary synthesis, because these studies directly informed the central review question. The appraisal was based on a qualitative framework adapted from the Newcastle–Ottawa Scale for non-randomized studies (11). The assessment focused on five domains: selection bias, outcome assessment, follow-up completeness, confounding, and overall risk of bias. Particular attention was paid to cohort assembly, clarity of transition definitions, completeness of long-term follow-up, and the potential for survivor selection. The resulting judgments are presented in Tables 3, 4.

**Table 3:** Risk of bias assessment of studies included in the primary synthesis of post-90-day functional trajectories after endovascular thrombectomy.

Study	Design	Selection bias	Outcome assessment	Follow-up completeness	Confounding	Overall risk	Key concern
Todo et al. (6)	Observational cohort	Moderate	Moderate	Moderate	High	Moderate to high	Limited reporting of cohort assembly and potential residual confounding; handling of loss to follow-up and post-90-day transition definitions should be interpreted cautiously.
Gunasekera et al. (7)	Observational cohort	Moderate	Moderate	High concern	High	High	Long-term functional assessment was based on a survivor subset, creating substantial risk of survivor selection bias and limiting generalizability of post-90-day recovery estimates.
Wang et al. (8)	Multicenter retrospective	Moderate	Low to moderate	Moderate	Moderate	Moderate	Retrospective design and residual confounding remain important limitations, but the large multicenter sample and explicit definition of 90-day to 1-year deterioration strengthen internal validity.

EVT, endovascular thrombectomy.

**Table 4:** Summary of risk-of-bias assessment for the studies included in the primary synthesis.

Study	Selection bias	Outcome assessment	Follow-up completeness	Confounding	Overall judgement
Todo et al. (6)	Some concerns/moderate	Some concerns/moderate	Some concerns/moderate	High risk/high concern	Moderate to high
Gunasekera et al. (7)	Some concerns/moderate	Some concerns/moderate	High risk/high concern	High risk/high concern	High risk/High concern
Wang et al. (8)	Some concerns/moderate	Low to moderate	Some concerns/moderate	Some concerns/moderate	Some concerns/moderate

Domain-level judgments are shown for selection bias, outcome assessment, follow-up completeness, confounding, and overall risk of bias. Risk-of-bias assessment was based on a qualitative framework adapted from the Newcastle-Ottawa Scale, with emphasis on cohort selection, ascertainment of long-term outcome, completeness of follow-up, and control of confounding.

### 2.8 Synthesis strategy

A conventional pooled meta-analysis was not performed because the directly eligible evidence base was extremely limited and both clinically and methodologically heterogeneous. Only three studies directly addressed post-90-day functional trajectory after EVT (6–8). These studies differed in several important respects, including follow-up anchors, denominator structure, stroke territory, and analytic focus. Two studies assessed change between 90 days and 1 year (6, 8), whereas one examined change between 3 months and 12 months (7). One study directly evaluated late deterioration (8), whereas the other two primarily informed delayed improvement (6, 7). In addition, one study was based on a survivor-restricted cohort (7), whereas another analyzed a large multicenter VBAO population (8).

Under these circumstances, any pooled quantitative estimate would likely have combined conceptually non-equivalent outcomes and produced a result that was statistically formal but clinically difficult to interpret. We therefore conducted a structured synthesis of the three directly eligible studies and a separate narrative contextual synthesis of the 11 retained supportive studies, grouped according to prognosis, durability of EVT benefit, etiologic or phenotypic heterogeneity, and rescue-strategy outcomes (9).

## 3 Results

### 3.1 Study selection

The database search yielded 283 records. After duplicate removal, 174 records underwent title/abstract screening, of which 121 were excluded. Fifty-three reports were sought for retrieval, and 1 report could not be obtained. As a result, 52 full-text reports were assessed for eligibility. Of these, 38 were excluded and 14 studies were retained in the final review, including 3 studies in the primary synthesis and 11 studies retained as supportive or discussion-only evidence. The study selection process is shown in Figure 1.

### 3.2 Characteristics of the primary synthesis studies

Three studies were included in the primary synthesis: Todo et al. (6), Gunasekera et al. (7), and Wang et al. (8). These studies were conducted in Japan, Australia, and China, respectively, and together covered EVT-treated populations with large-vessel occlusion ischemic stroke, basilar artery occlusion (BAO), and vertebrobasilar artery occlusion (VBAO). All three were observational studies, although their designs and analytic objectives differed. Todo et al. (6) examined long-term outcome beyond 90 days in EVT-treated ischemic stroke, Gunasekera et al. (7) evaluated functional change between 3 months and 12 months in surviving patients with BAO, and Wang et al. (8) assessed deterioration between 90 days and 1 year in a large multicenter VBAO cohort.

The directly eligible studies were heterogeneous in several clinically relevant aspects. Follow-up anchors were not fully aligned, with two studies evaluating change between 90 days and 1 year (6, 8) and one comparing 3-month and 12-month outcomes (7). Denominator structure also varied, most notably because Gunasekera et al. (7) analyzed long-term survivors with available follow-up. In addition, the studies did not assess an identical trajectory construct: Todo et al. (6) and Gunasekera et al. (7) primarily informed delayed functional improvement, whereas Wang et al. (8) directly addressed late functional deterioration. This combination of heterogeneity in population, follow-up, and outcome definition limited direct quantitative pooling and supported the use of structured primary synthesis. The characteristics of the primary synthesis studies are summarized in Table 1.

### 3.3 Delayed functional improvement beyond 90 days

#### 3.3.1 Todo et al.

Todo et al. (6) provided core direct evidence that functional outcome after EVT may continue to evolve between 90 days and 1 year. In the harmonized extraction for the present review, 46 of 268 patients (17.2%) were classified as having delayed improvement, 84 (31.3%) as having deteriorated, and 138 (51.5%) as having remained stable over this interval. These findings suggest that the conventional 90-day assessment may underestimate eventual recovery in a clinically meaningful minority of EVT-treated patients.

#### 3.3.2 Gunasekera et al.

Gunasekera et al. (7) extended this concept to a posterior circulation cohort undergoing EVT for BAO. Among surviving patients with available long-term follow-up, 21 of 51 (41.2%) improved between 3 months and 12 months, 8 (15.7%) deteriorated, and 22 (43.1%) remained stable. This study supports the possibility of continued recovery beyond the conventional short-term endpoint in selected posterior circulation survivors. However, because the analysis was restricted to survivors with available follow-up, the magnitude of delayed recovery should be interpreted cautiously in light of possible survivor selection.

#### 3.3.3 Interim interpretation

Taken together, these two studies indicate that recovery after EVT is not invariably complete by 90 days (6, 7). Rather, a subset of patients continues to improve during later follow-up. At the same time, the apparent magnitude of delayed improvement varies considerably across studies and is likely influenced by study design, vascular territory, denominator structure, and follow-up selection. The main findings related to delayed functional improvement are summarized in Table 2.

### 3.4 Late functional deterioration beyond 90 days

#### 3.4.1 Wang et al.

Late functional deterioration after the conventional endpoint was directly evaluated by Wang et al. (8). In this multicenter retrospective study of 1,342 patients with VBAO treated with EVT, deterioration from 90 days to 1 year was defined as a worsening of at least 1 point in mRS. Overall, 288 patients deteriorated and 1,054 remained stable, corresponding to a deterioration rate of 21.5% (8). After multivariable adjustment, female sex, older age, longer door-to-puncture time, and four or more thrombectomy passes were independently associated with late deterioration, whereas better reperfusion was protective (8). In the harmonized extraction, the reported estimates included female sex (OR 1.425), age per year (OR 1.016), door-to-puncture time (OR 1.001), and ≥4 thrombectomy passes (OR 3.765), with better reperfusion associated with lower odds of worsening.

#### 3.4.2 Bidirectional trajectory interpretation

The findings of Wang et al. (8) broaden the interpretation of long-term post-EVT outcome from delayed recovery alone to a bidirectional trajectory model. Importantly, the two studies primarily addressing delayed recovery also reported subgroups of patients who deteriorated during later follow-up (6, 7). Thus, the directly eligible literature indicates that post-90-day functional status after EVT does not follow a single direction: later recovery occurs, but later decline also occurs. The key findings related to late functional deterioration are summarized in Table 2.

### 3.5 Supportive long-term outcome evidence

#### 3.5.1 Prognostic and risk-stratification studies

A first group of supportive studies consisted of prognostic and risk-stratification analyses. These studies examined factors such as coagulation-related measures, renal impairment, symptom phenotypes, age-related differences, and longer-term mortality, thereby demonstrating that late outcome after EVT is clinically heterogeneous. Their value in the present review is explanatory rather than primary: they help clarify why long-term outcome may differ across patients, but they do not determine whether individual patients improved, remained stable, or deteriorated after 90 days. Accordingly, they inform interpretation of long-term heterogeneity without providing direct evidence of post-90-day functional transition.

#### 3.5.2 Treatment-effect durability studies

A second group consisted of treatment-effect durability studies. Representative examples included the 1-year follow-up of ATTENTION (12), the 2-year follow-up of MR CLEAN-LATE (13), and the 12-month outcomes of TENSION (14). These studies are important because they demonstrate that long-term follow-up after EVT is clinically meaningful and that treatment benefit may persist beyond the conventional 90-day endpoint. However, durability of treatment effect is not equivalent to within-patient trajectory analysis. A study may show sustained EVT benefit at 1 year or 2 years without clarifying whether individual patients improved, remained stable, or deteriorated after the 90-day assessment. For this reason, these studies were retained as supportive contextual evidence but were not eligible for the primary synthesis.

#### 3.5.3 Etiologic/phenotypic heterogeneity studies

A third group addressed etiologic or phenotypic heterogeneity. These studies suggested that long-term outcome after EVT varies according to stroke mechanism, baseline phenotype, or subgroup characteristics. Such findings are relevant to the present review because they reinforce the notion that long-term post-EVT recovery is not uniform. However, these studies focused on between-group differences in later outcome rather than on direct within-patient transition beyond 90 days.

#### 3.5.4 Rescue-strategy studies

A fourth group comprised rescue-strategy or technique-specific studies. These reports informed the long-term prognosis of particular treatment subgroups, such as patients undergoing rescue stenting or angioplasty. Their contribution was therefore contextual rather than primary. They help characterize longer-term EVT-related outcomes in selected procedural settings, but they do not address the natural history of post-90-day functional change in the broader EVT population.

#### 3.5.5 Synthesis of supportive evidence

**Figure 2:**
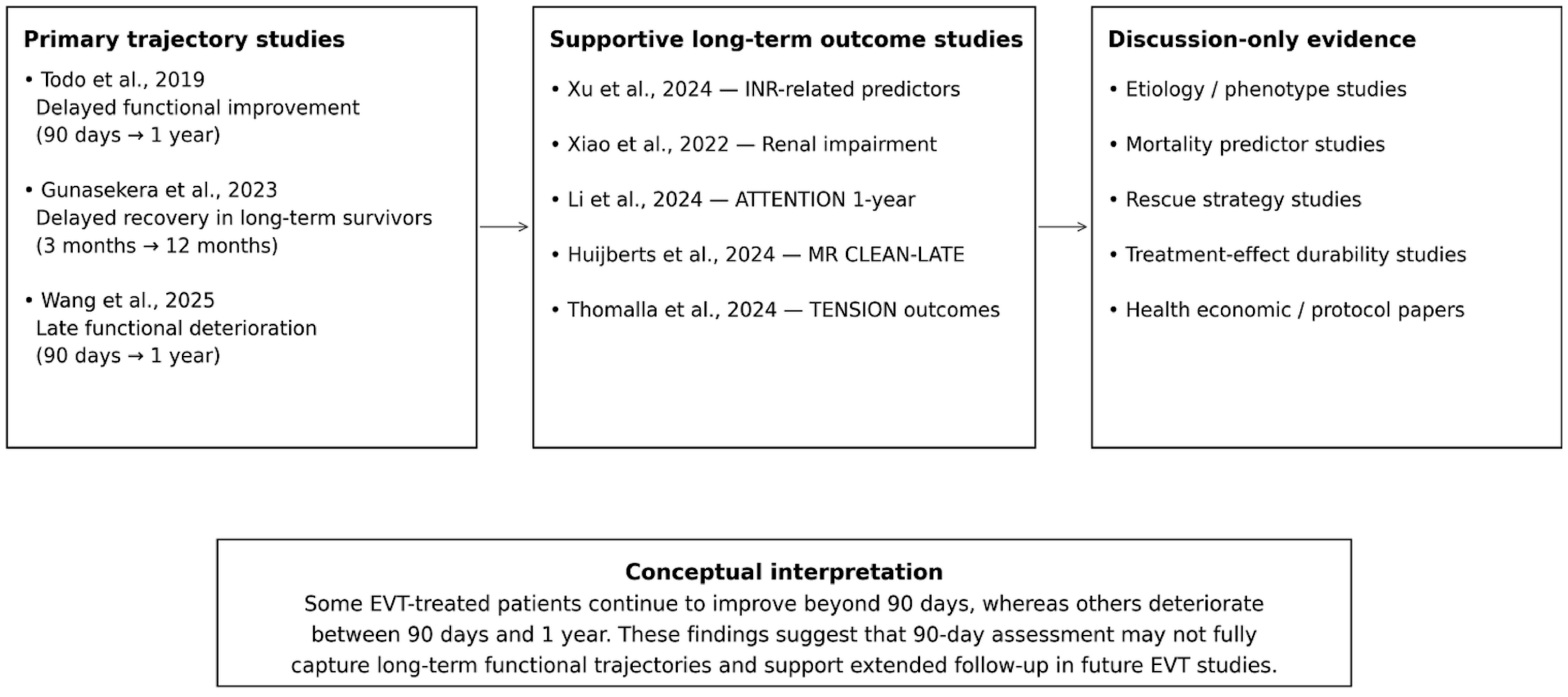
Evidence map of retained studies on functional trajectories beyond 90 days after endovascular thrombectomy. The figure distinguishes directly eligible post-90-day trajectory studies from supportive long-term outcome studies retained for contextual interpretation. EVT, endovascular thrombectomy; INR, international normalized ratio.

Taken together, the supportive literature indicates that long-term outcome after EVT is clinically important, heterogeneous, and increasingly well documented. However, it more often describes late outcome status or durability of treatment effect than explicit late within-patient transition. This distinction helps explain the apparent mismatch between the growing number of long-term EVT studies and the small number of studies directly eligible for the present primary synthesis. In other words, the broader literature on long-term EVT outcome is more extensive than the literature directly addressing post-90-day functional trajectory. The hierarchy of retained supportive studies is summarized in Supplementary Table S1, the conceptual structure of the retained evidence is illustrated in Figure 2 and, where appropriate, Table 5, and full-text reports excluded after eligibility assessment with specific reasons are listed in Supplementary Table S2.

**Table 5:** Selected study-level outcome matrix of functional trajectories beyond 90 days after endovascular thrombectomy.

Study	Population	Follow-up points	Delayed improvement	Late deterioration	Long-term outcome	Role in review
Todo et al. (6)	EVT-treated acute ischemic stroke	90 days → 1 year	●	—	●	Primary synthesis
Gunasekera et al. (7)	Basilar artery occlusion treated with EVT	3 months → 12 months	●	—	●	Primary synthesis (conditional)
Wang et al. (8)	Vertebrobasilar artery occlusion treated with EVT	90 days → 1 year	—	●	●	Primary synthesis (late deterioration)
Xu et al. (15)	Vertebrobasilar artery occlusion treated with EVT	90 days and 1 year	—	—	●	Adjacent evidence
Xiao et al. (16)	Vertebrobasilar artery occlusion treated with EVT	3 months and 1 year	—	—	●	Discussion-only
Li et al. (12)	Basilar artery occlusion (ATTENTION 1-year follow-up)	90 days and 1 year	—	—	●	Discussion-only
Huijberts et al. (13)	Late-window anterior circulation LVO (MR CLEAN-LATE)	90 days and 2 years	—	—	●	Discussion-only
Thomalla et al. (14)	Large-core anterior circulation stroke (TENSION)	90 days and 12 months	—	—	●	Discussion-only

Filled circles indicate direct evidence for the specified post-90-day domain. Dashes indicate that long-term outcomes were reported, but within-patient post-90-day functional transition was not directly assessed.

AIS, acute ischemic stroke; EVT, endovascular thrombectomy; LVO, large-vessel occlusion.

### 3.6 Risk of bias of the primary synthesis studies

#### 3.6.1 Todo

Todo et al. (6) was judged to have moderate selection bias, moderate concerns regarding outcome assessment and follow-up completeness, high concern for confounding, and an overall moderate-to-high risk of bias (11). The main concerns were limited reporting of cohort assembly, residual confounding, and uncertainty regarding the detailed handling of follow-up transition definitions.

#### 3.6.2 Gunasekera

Gunasekera et al. (7) was judged to have moderate selection bias, moderate concern regarding outcome assessment, high concern regarding follow-up completeness, high concern for confounding, and an overall high risk of bias (11). The principal limitation was survivor selection, as long-term assessment was based on a survivor subset with available follow-up.

#### 3.6.3 Wang

Wang et al. (8) was judged to have moderate selection bias, low-to-moderate concern regarding outcome assessment, moderate concern regarding follow-up completeness and confounding, and an overall moderate risk of bias (11). Although the retrospective design leaves room for residual confounding, the large multicenter sample and explicit definition of deterioration strengthen internal validity relative to the other directly eligible reports.

#### 3.6.4 Overall interpretation

Overall, the directly eligible literature is informative but methodologically limited. The available evidence supports the credibility of both delayed improvement and late deterioration after EVT; however, the precision and generalizability of current estimates remain constrained by observational design, heterogeneity, incomplete alignment of follow-up structures, and follow-up-related bias (6–8, 11). The domain-level assessments are summarized in Tables 3, 4.

## 4 Discussion

### 4.1 Principal findings

This systematic review suggests that functional outcome after endovascular thrombectomy (EVT) is not invariably fixed at 90 days. Across the three directly eligible studies, two clinically important observations emerged. First, a proportion of patients continued to improve functionally beyond the conventional 90-day endpoint (6, 7). Second, a meaningful subset deteriorated after apparently established short-term outcome, including in a large multicenter vertebrobasilar artery occlusion (VBAO) cohort (8). Taken together, these findings support interpreting post-90-day outcome after EVT as a trajectory rather than as a single late cross-sectional measurement.

At the same time, the directly eligible literature remains limited. Only three studies directly examined within-patient functional change beyond the conventional endpoint, and they differed in follow-up anchor, study population, denominator structure, and trajectory definition (6–8). The evidence base is therefore small, but conceptually important. Its value lies less in numerical precision than in showing that the tacit assumption of a universally fixed 90-day outcome after EVT is not always justified.

### 4.2 The real message: 90-day outcome is informative, but not always definitive

The central message of this review is not that the 90-day endpoint should be abandoned, but that it should be interpreted with greater conceptual precision. The 90-day mRS remains methodologically robust, clinically interpretable, and historically grounded in the landmark EVT trials and subsequent guideline frameworks (1–5). However, a conventional endpoint is not equivalent to biological finality. The directly eligible evidence synthesized here suggests that some patients continue to recover after 90 days, whereas others worsen despite an apparently established short-term result (6–8). Thus, the distinction between endpoint convention and recovery finality is fundamental: the former is a methodological standard, whereas the latter is a biological and clinical question.

This distinction matters for both interpretation and study design. If 90-day status is treated as definitively final, later recovery may be underestimated and later decline may be overlooked. Conversely, if longer-term outcome is approached through a trajectory framework, later functional change becomes a phenomenon to be studied explicitly rather than residual variation to be ignored. The present review therefore supports a more nuanced view: 90-day outcome remains informative and should remain central, but it should not necessarily be regarded as the final word on recovery after EVT (1–8).

### 4.3 Delayed improvement as a clinically relevant phenomenon

The evidence for delayed improvement is clinically relevant, even if currently limited. Todo et al. (6) showed that successful reperfusion with EVT was associated with beneficial long-term outcome beyond 90 days, suggesting that recovery may continue after the conventional endpoint. Gunasekera et al. (7) reported that a substantial proportion of surviving patients with basilar artery occlusion improved between 3 months and 12 months after thrombectomy. Although the absolute estimates differed, the overall signal was consistent: post-90-day improvement is real and should not be dismissed as anecdotal.

This phenomenon may be particularly relevant in several clinical contexts. Patients with severe early deficits may require a longer recovery window before meaningful functional gains become evident. Posterior circulation stroke may also follow a recovery course that is not fully captured at 90 days, particularly among survivors with incomplete early recovery (7). In addition, patients whose early disability reflects a combination of acute neurological injury, prolonged hospitalization, deconditioning, and delayed rehabilitation engagement may demonstrate meaningful improvement only after the conventional trial endpoint.

Several mechanisms may plausibly contribute to delayed improvement. Neurological recovery may continue beyond the early post-stroke phase, particularly when reperfusion has limited infarct progression but functional reintegration remains delayed. Rehabilitation-related gains may accumulate over time, especially in patients with severe early disability but residual recovery potential. Delayed adaptation to disability, improved mobility, better medical stabilization, and resolution of post-acute complications may also contribute to functional improvement that is not fully captured at 90 days. Although the present review cannot formally disentangle these mechanisms, the available evidence supports the view that later recovery after EVT is both biologically plausible and clinically meaningful (6, 7).

Delayed improvement should not, however, be overstated. The magnitude of later recovery varied substantially across the directly eligible studies (6, 7), and part of this variation likely reflects methodological rather than biological differences. In particular, Gunasekera et al. (7) evaluated long-term survivors with available follow-up, which may enrich the denominator for patients with continued recovery potential. Even so, the existence of delayed improvement across different settings argues against viewing 90-day outcome as universally final. At the same time, this conclusion should be interpreted cautiously because the available evidence for delayed improvement is derived from a small number of observational studies, including one survivor-restricted cohort, and is therefore vulnerable to selection bias and limited generalizability.

### 4.4 Late deterioration as an equally important long-term phenomenon

A focus on later recovery alone is insufficient. The evidence synthesized in this review also indicates that late deterioration is an important component of long-term outcome after EVT. Wang et al. (8) showed that 21.5% of patients with VBAO experienced worsening in mRS between 90 days and 1 year, indicating that later decline is clinically relevant rather than exceptional. This finding is particularly important because it shifts the field beyond a recovery-centered narrative toward a bidirectional model of post-90-day outcome.

The clinical implications are substantial. A favorable 90-day result does not necessarily guarantee long-term stability. Patients may subsequently deteriorate because of recurrent vascular events, progressive frailty, delayed medical complications, incomplete neurological stabilization, or treatment-related and disease-related factors that are not fully expressed at the short-term endpoint. The predictors identified by Wang et al. (8)—including older age, female sex, longer door-to-puncture time, and multiple thrombectomy passes—suggest that later decline may be shaped by both patient-level vulnerability and procedural complexity.

This broader perspective also helps reinterpret the delayed-recovery studies. Todo et al. (6) and Gunasekera et al. (7) were primarily relevant because they documented later improvement, yet both also included subsets of patients who worsened during later follow-up. This internal pattern reinforces the central point of the review: post-90-day functional outcome after EVT is not unidirectional. Some patients improve, some remain stable, and some deteriorate. Any framework that considers only recovery captures only part of the longer-term clinical picture.

From an interpretive standpoint, late deterioration is especially important because it complicates how 90-day trial results are translated into prognosis. If later worsening occurs in a clinically meaningful subset, short-term success should not automatically be interpreted as long-term stability. This does not diminish the value of the 90-day endpoint, but it does suggest that later decline deserves greater attention in long-term EVT reporting and post-stroke follow-up (8). However, the current evidence for late deterioration is also limited by observational design and residual confounding, even though the multicenter Wang et al. study provides comparatively stronger direct evidence for this trajectory direction than is currently available for delayed improvement.

### 4.5 Why the long-term EVT literature is broader than the post-90-day trajectory literature

One of the clearest insights from this review is that the literature on long-term EVT outcome is substantially broader than the literature directly addressing post-90-day trajectory. Many contemporary studies now report 1-year or even 2-year outcomes after EVT, including randomized or comparative follow-up analyses in basilar artery occlusion, late-window stroke, and large-infarct populations (12–14). This growing body of work confirms that longer-term follow-up is both feasible and clinically relevant.

However, these studies are conceptually distinct from direct trajectory studies. A report showing that EVT benefit persists at 1 year does not necessarily indicate whether individual patients improved, remained stable, or deteriorated after 90 days. Similarly, a 2-year outcome analysis may demonstrate sustained treatment effect or durable between-group differences without directly examining within-patient functional transition beyond the conventional endpoint (12–14). In other words, reporting a later endpoint is not the same as analyzing a later trajectory.

This distinction explains why supportive evidence was broader than primary evidence in the present review. Studies such as the 1-year follow-up of ATTENTION, the 2-year follow-up of MR CLEAN-LATE, and the 12-month outcomes of TENSION show that long-term EVT outcomes matter and that later follow-up can refine understanding of treatment benefit (12–14). Yet these studies do not answer the central question posed here: what happens to individual patients after the 90-day assessment? Do they improve, remain stable, or decline?

The broader long-term EVT literature is therefore highly relevant, but in a contextual rather than primary sense. It confirms the importance of later follow-up, highlights heterogeneity of late outcome, and supports the clinical value of long-term assessment. At the same time, it does not resolve the central evidence gap identified in this review, namely the scarcity of studies directly quantifying post-90-day within-patient transition. This distinction is essential: having long-term data is not the same as understanding long-term functional trajectory.

### 4.6 Implications for future research

The present review has several implications for future research. First, studies extending follow-up beyond 90 days should move beyond simply reporting a later mRS distribution. Reporting 1-year outcome alone is insufficient if the aim is to understand whether recovery continues, stabilizes, or deteriorates after the conventional endpoint. Future studies should therefore distinguish explicitly between delayed improvement, functional stability, and late deterioration.

Second, standardized post-90-day definitions are needed. The directly eligible studies differed in follow-up anchors and operationalization of transition (6–8), which limited comparability and precluded conventional meta-analysis. A more unified approach to defining later recovery and later deterioration would improve interpretability across studies and permit more robust synthesis in the future.

Third, multicenter trajectory-based designs are needed. Large, prospectively followed EVT cohorts with standardized follow-up procedures would allow investigators to model post-90-day change more rigorously, identify predictors of delayed recovery and late worsening, and determine whether trajectory patterns differ by vascular territory, baseline severity, reperfusion quality, or post-acute care factors. Such work could build on the signal already identified in the directly eligible studies and complement the broader long-term outcome literature (6–8, 12–14).

Finally, future studies should consider integrating post-acute factors more explicitly. If post-90-day outcome is dynamic, variables such as rehabilitation intensity, recurrent vascular events, medical complications, frailty, and social support may become increasingly relevant after the acute phase. A trajectory-based framework may therefore be better suited than conventional endpoint reporting to capture these influences.

### 4.7 Clinical implications

The findings of this review also have practical clinical implications. Clinicians should be cautious about treating 90-day outcome as a definitively final endpoint in all EVT-treated patients. Some patients, particularly those with incomplete early recovery, may improve meaningfully during longer follow-up (6, 7). Others may deteriorate after an apparently favorable short-term result (8). This means that the prognostic discussion at 90 days may sometimes need to remain open rather than final.

Longer observation may therefore affect counseling and prognosis communication. For patients with residual recovery potential, clinicians may wish to frame the 90-day result as an important milestone rather than the absolute end of recovery. For patients at higher risk of late decline, longer-term monitoring may help identify clinical worsening that would otherwise be missed if follow-up were effectively truncated at 90 days. In this sense, post-90-day follow-up is not only of research interest; it may also influence how clinicians communicate uncertainty, organize longer-term care, and interpret outcome after EVT (6–8).

These findings may also have implications for follow-up planning and rehabilitation referral after EVT. If functional status at 90 days does not always reflect the final course of recovery, some patients with persistent but potentially reversible disability may benefit from continued reassessment beyond the conventional short-term endpoint. In practical terms, a trajectory-based perspective may support more individualized follow-up scheduling, encourage continued rehabilitation referral in selected patients with residual recovery potential, and prompt closer surveillance in patients considered vulnerable to later decline. Although current evidence remains limited, the present review suggests that the 90-day visit should not always be treated as the final opportunity to reassess recovery after EVT.

### 4.8 Limitations

Several limitations should be acknowledged. First, only three studies directly addressed post-90-day functional trajectory after EVT (6–8). This limited evidence base precluded conventional pooled meta-analysis and instead required a structured synthesis approach aligned with SWiM principles (9). Second, the directly eligible studies were heterogeneous in follow-up anchor, denominator structure, study population, and trajectory definition, which reduced comparability and limited the interpretability of any pooled quantitative estimate (6–8). Third, all directly eligible studies were observational, and the risk-of-bias assessment identified important concerns related to confounding, follow-up completeness, and cohort selection (11). Survivor selection was particularly relevant in the BAO study by Gunasekera et al. (7). Fourth, the supportive studies retained in the review were contextual rather than primary and should not be interpreted as direct evidence of delayed recovery or late deterioration after 90 days. Overall, the certainty of the current evidence is low. This reflects the small number of directly eligible studies, their observational design, heterogeneity in vascular territory, follow-up structure, denominator definition, and transition construct, as well as important concerns regarding confounding and selection bias. The findings of the present review should therefore be regarded as hypothesis-generating rather than as definitive estimates of the frequency or determinants of post-90-day functional change after EVT.

Importantly, the strength of inference differs across trajectory directions. Evidence for delayed improvement is currently supported by fewer and more selective observational datasets, whereas evidence for late deterioration is somewhat more directly quantified but remains subject to residual confounding and heterogeneity. Accordingly, neither trajectory direction should yet be interpreted as supported by high-certainty evidence.

### 4.9 Final interpretive statement

In summary, the available literature supports the view that functional trajectory after EVT can extend beyond 90 days and may proceed in either direction (6–8). Although the directly eligible evidence remains limited, it identifies an under-recognized but clinically meaningful concept: the conventional short-term endpoint does not always capture the final course of recovery after EVT. Long-term EVT outcome should therefore be interpreted through a trajectory lens, with explicit attention to delayed improvement, functional stability, and late deterioration rather than reliance on later endpoint status alone.

## 5 Conclusion

Current evidence suggests that 90-day outcome after endovascular thrombectomy may not always represent final recovery. Both delayed functional improvement and late functional deterioration appear possible beyond the conventional short-term endpoint, but the certainty of current evidence remains low. These findings should therefore be regarded as hypothesis-generating and support the need for prospective, standardized, trajectory-based follow-up after EVT.

## Data Availability

This study is a systematic review based on previously published literature. No new dataset was generated for this study. The data supporting the findings of this review are available in the cited articles and in the tables and Supplementary material provided in this manuscript.

## References

[1] Berkhemer OA Fransen PSS Beumer D van den Berg LA Lingsma HF Yoo AJ . A randomized trial of intraarterial treatment for acute ischemic stroke. N Engl J Med. (2015) 372:11–20. doi: 10.1056/NEJMoa1411587 25517348

[2] Goyal M Demchuk AM Menon BK Eesa M Rempel JL Thornton J . Randomized assessment of rapid endovascular treatment of ischemic stroke. N Engl J Med. (2015) 372:1019–30. doi: 10.1056/NEJMoa1414905, 25671798

[3] Saver JL Goyal M Bonafe A Diener HC Levy EI Pereira VM . Stent-retriever thrombectomy after intravenous t-PA vs. t-PA alone in stroke. N Engl J Med. (2015) 372:2285–95. doi: 10.1056/NEJMoa1415061, 25882376

[4] Campbell BCV Mitchell PJ Kleinig TJ Dewey HM Churilov L Yassi N . Endovascular therapy for ischemic stroke with perfusion-imaging selection. N Engl J Med. (2015) 372:1009–18. doi: 10.1056/NEJMoa1414792, 25671797

[5] Powers WJ Rabinstein AA Ackerson T Adeoye OM Bambakidis NC Becker K . Guidelines for the early management of patients with acute ischemic stroke: 2019 update to the 2018 guidelines for the early management of acute ischemic stroke: a guideline for healthcare professionals from the American Heart Association/American Stroke Association. Stroke. (2019) 50:e344–418. doi: 10.1161/STR.0000000000000211, 31662037

[6] Todo K Sakai N Imamura H Yamagami H Adachi H Kono T . Successful reperfusion with endovascular therapy has beneficial effects on long-term outcome beyond 90 days. Cerebrovasc Dis. (2019) 47:127–34. doi: 10.1159/000499190, 30965319

[7] Gunasekera L Mitchell P Dowling RJ Bush S Yan B. Functional recovery continues beyond 3 months post-basilar artery thrombectomy: a retrospective cohort study. CNS Neurosci Ther. (2023) 29:2171–6. doi: 10.1111/cns.14182, 36942501 PMC10352879

[8] Wang L Sun W Ping L Chen X Fu Z Liu J . Predictors of functional deterioration from 90 days to 1 year after endovascular treatment for vertebrobasilar artery occlusion: a multicenter retrospective study. Front Neurol. (2025) 16:1671672. doi: 10.3389/fneur.2025.1671672, 41255785 PMC12621454

[9] Campbell M McKenzie JE Sowden A Katikireddi SV Brennan SE Ellis S . Synthesis without meta-analysis (SWiM) in systematic reviews: reporting guideline. BMJ. (2020) 368:l6890. doi: 10.1136/bmj.l6890, 31948937 PMC7190266

[10] Page MJ McKenzie JE Bossuyt PM Boutron I Hoffmann TC Mulrow CD . The PRISMA 2020 statement: an updated guideline for reporting systematic reviews. BMJ. (2021) 372:n71. doi: 10.1136/bmj.n71, 33782057 PMC8005924

[11] Wells GA Shea B O’Connell D Peterson J Welch V Losos M . The Newcastle-Ottawa scale (NOS) for assessing the quality of nonrandomised studies in meta-analyses. Ottawa: Ottawa Hospital Research Institute. (2011). Available online at: https://www.ohri.ca/programs/clinical_epidemiology/oxford.asp (Accessed July 5, 2026).

[12] Li R Tao C Sun J Zhang C Xu P Yin Y . Endovascular vs medical management of acute basilar artery occlusion: a secondary analysis of a randomized clinical trial. JAMA Neurol. (2024) 81:1043–50. doi: 10.1001/jamaneurol.2024.2652, 39186280 PMC11348088

[13] Huijberts I Pinckaers FME Olthuis SGH van Kuijk SMJ Postma AA Boogaarts HD . Collateral-based selection for endovascular treatment of acute ischaemic stroke in the late window (MR CLEAN-LATE): 2-year follow-up of a phase 3, multicentre, open-label, randomised controlled trial in the Netherlands. Lancet Neurol. (2024) 23:893–900. doi: 10.1016/S1474-4422(24)00228-X, 38909624

[14] Thomalla G Fiehler J Subtil F Bonekamp S Aamodt AH Fuentes B . Endovascular thrombectomy for acute ischaemic stroke with established large infarct (TENSION): 12-month outcomes of a multicentre, open-label, randomised trial. Lancet Neurol. (2024) 23:883–92. doi: 10.1016/S1474-4422(24)00278-3, 39074480

[15] Xu Y Huang Z Zhang P Zhong J Zhang W Hu M . Effect of INR on outcomes of endovascular treatment for acute vertebrobasilar artery occlusion. Transl Stroke Res. (2024) 15:916–924. doi: 10.1007/s12975-023-01176-y 37442918

[16] Xiao L Gu M Lu Y Xu P Wang J Lan W . Influence of renal impairment on clinical outcomes after endovascular recanalization in vertebrobasilar artery occlusions. J Neurointerv Surg. (2022) 14:1077–1083. doi: 10.1136/neurintsurg-2021-018003 34853176

