# Supplementary material for: Functional trajectories beyond 90 days after endovascular thrombectomy: a systematic review with primary synthesis and supportive narrative evidence
Source: Front Neurol. 2026 Aug 3;17:1847994. doi: 10.3389/fneur.2026.1847994 (PMC13482335; doi:10.3389/fneur.2026.1847994)
Supplement: Supplementary file 1 [file Supplementary_file_1.docx]

**Supplementary Appendix 1. Search strategy and review procedures**

**1. Review objective**

This systematic review was designed to identify studies examining functional trajectories beyond the conventional 90-day endpoint after endovascular thrombectomy (EVT), with particular emphasis on delayed functional improvement, late functional deterioration, and other long-term outcome patterns relevant to the interpretation of post-90-day recovery.

**2. Databases searched**

The literature search was conducted in the following electronic databases:

- PubMed
- Embase
- Web of Science Core Collection
- Cochrane Library

All databases were searched from inception to the final search date.

**3. Final search date**

The final search was conducted on 14 April 2026.

**4. Search framework**

The search strategy combined terms from four broad conceptual domains:

- Stroke / vascular occlusion terms, including ischemic stroke, large-vessel occlusion, basilar artery occlusion, vertebrobasilar artery occlusion, and related cerebrovascular terms.
- EVT / thrombectomy terms, including endovascular thrombectomy, mechanical thrombectomy, endovascular treatment, reperfusion therapy, and related procedural terms.
- Longer-term follow-up terms, including 90 days, 3 months, 1 year, 12 months, long-term outcome, extended follow-up, and related time-structure expressions.
- Functional transition terms, including delayed recovery, delayed functional improvement, functional deterioration, late deterioration, and related trajectory-oriented concepts.

The search strategy was designed to identify both directly eligible post-90-day trajectory studies and broader long-term EVT outcome studies that might contribute supportive contextual evidence.

**5. Search limits and restrictions**

- Language restriction: English-language records were retained in the final screened set.
- Document type restriction: Article-type records were retained in the final screened set.
- Date restriction: None; all databases were searched from inception to the final search date.
- Species restriction: No explicit animal filter was applied in the search strategy, but screening was restricted to human clinical studies.
- Grey literature: No formal grey-literature database search was undertaken.
- Reference checking: Reference lists of eligible full-text articles were manually reviewed to identify potentially relevant additional studies.

**6. Database-specific final yield**

The final harmonized search yield used for the review was as follows:

- PubMed: 123 records
- Embase: 78 records
- Web of Science Core Collection: 53 records
- Cochrane Library: 29 records

Total records identified from databases: 283. Full database-specific search strategies are provided in Section 14.

**7. Records management and deduplication**

All retrieved records were exported into EndNote for reference management and duplicate handling. After duplicate removal, 109 records were removed as duplicates, leaving 174 records for title/abstract screening.

**8. Screening and study selection**

Study selection was performed in a hierarchical manner to preserve the distinction between directly relevant post-90-day trajectory evidence and broader long-term contextual evidence.

The final screening and eligibility process was as follows:

- Records identified from databases: 283
- Duplicates removed: 109
- Records screened after deduplication: 174
- Records excluded after title/abstract screening: 121
- Reports sought for retrieval: 53
- Reports not retrieved: 1
- Full-text reports assessed for eligibility: 52
- Full-text reports excluded, with reasons: 38
- Studies retained in the review: 14
- Included in primary synthesis: 3
- Retained as supportive / discussion-only evidence: 11

**9. Eligibility hierarchy**

Studies assessed at full text were classified into three hierarchical groups.

**9.1 Primary synthesis studies**

Primary synthesis studies were defined as studies directly assessing within-patient functional transition beyond the conventional 90-day endpoint after EVT. Eligible studies in this category explicitly evaluated whether patients improved, remained stable, or deteriorated between 90 days and a later follow-up point, or between 3 months and a later time point when the analysis clearly addressed post-conventional-endpoint change.

**9.2 Supportive / discussion-only evidence**

Supportive or discussion-only evidence comprised studies reporting longer-term EVT outcomes without explicit analysis of within-patient post-90-day transition. These studies were retained when they contributed clinically relevant contextual evidence regarding prognosis, durability of EVT benefit, etiologic or phenotypic heterogeneity, or outcomes associated with rescue strategies.

**9.3 Excluded full-text reports**

Full-text reports were excluded when they did not directly address the review question or did not meet the prespecified eligibility framework. The reasons for exclusion were classified as follows:

- Not a direct post-90-day functional trajectory study (n = 11)
- Prognostic factor study only (n = 7)
- Treatment-effect durability / long-term efficacy study only (n = 2)
- Rescue strategy / technique-specific study (n = 5)
- Review / protocol / guideline / health economic study (n = 6)
- Wrong population / not EVT-specific (n = 6)
- Other reasons (n = 1)

A full list of excluded full-text reports and their specific reasons for exclusion is provided in Supplementary Table S2.

**10. Data extraction**

Data extraction was performed using structured extraction fields.

For studies included in the primary synthesis, extracted variables included:

- study design
- country / region
- study population
- occlusion territory
- sample size
- follow-up points
- post-90-day trajectory domain
- key operational definition
- quantitative findings
- interpretive role in the current review

For studies retained as supportive evidence, extracted variables included:

- study type
- population / stroke type
- follow-up structure
- main focus
- reason not included in the primary synthesis
- suggested interpretive role in the manuscript

**11. Reviewer process and conflict resolution**

Initial screening, eligibility assessment, and extraction were conducted using structured review forms by the first author. Any uncertainties regarding study classification or interpretive role were resolved through discussion with the corresponding author.

**12. Protocol and registration**

This review was not prospectively registered. However, the review question, eligibility framework, and structured synthesis approach were prespecified by the authors before full-text synthesis.

**13. Synthesis approach**

Because only three studies directly assessed post-90-day within-patient functional trajectory after EVT, and because these studies differed in vascular territory, follow-up anchor, denominator structure, and transition construct, a conventional pooled meta-analysis was not performed. Instead, the review used a structured primary synthesis of the directly eligible studies and a separate supportive narrative contextual synthesis of the retained long-term outcome literature.

**14. Exact database-specific search strategies**

The following strategies represent the final database-specific core search strings. English-language and article-type restrictions were applied at the database interface level where applicable, and the final record counts reported in the manuscript reflect the post-filter search yield.

**14.1 PubMed**

```
(
  "Stroke"[Mesh]
  OR "Brain Ischemia"[Mesh]
  OR "acute ischemic stroke"[Title/Abstract]
  OR "ischemic stroke"[Title/Abstract]
  OR "large vessel occlusion"[Title/Abstract]
  OR LVO[Title/Abstract]
  OR "basilar artery occlusion"[Title/Abstract]
  OR BAO[Title/Abstract]
  OR "vertebrobasilar occlusion"[Title/Abstract]
  OR VBAO[Title/Abstract]
)
AND
(
  "Thrombectomy"[Mesh]
  OR "endovascular thrombectomy"[Title/Abstract]
  OR "mechanical thrombectomy"[Title/Abstract]
  OR EVT[Title/Abstract]
  OR "endovascular treatment"[Title/Abstract]
  OR "endovascular therapy"[Title/Abstract]
  OR "stent retriever"[Title/Abstract]
  OR "aspiration thrombectomy"[Title/Abstract]
)
AND
(
  "Treatment Outcome"[Mesh]
  OR "Follow-Up Studies"[Mesh]
  OR "long-term"[Title/Abstract]
  OR "1 year"[Title/Abstract]
  OR "12 month*"[Title/Abstract]
  OR "beyond 90 days"[Title/Abstract]
  OR "90 days to 1 year"[Title/Abstract]
  OR "delayed recovery"[Title/Abstract]
  OR "delayed functional improvement"[Title/Abstract]
  OR "late functional deterioration"[Title/Abstract]
  OR "functional trajectory"[Title/Abstract]
  OR "modified Rankin Scale"[Title/Abstract]
  OR mRS[Title/Abstract]
)
```

**14.2 Embase**

```
(
  'stroke'/exp
  OR 'brain ischemia'/exp
  OR 'acute ischemic stroke':ti,ab
  OR 'ischemic stroke':ti,ab
  OR 'large vessel occlusion':ti,ab
  OR lvo:ti,ab
  OR 'basilar artery occlusion':ti,ab
  OR bao:ti,ab
  OR 'vertebrobasilar occlusion':ti,ab
  OR vbao:ti,ab
)
AND
(
  'thrombectomy'/exp
  OR 'endovascular thrombectomy':ti,ab
  OR 'mechanical thrombectomy':ti,ab
  OR evt:ti,ab
  OR 'endovascular treatment':ti,ab
  OR 'endovascular therapy':ti,ab
  OR 'stent retriever':ti,ab
  OR 'aspiration thrombectomy':ti,ab
)
AND
(
  'treatment outcome'/exp
  OR 'follow up'/exp
  OR 'long term follow up'/exp
  OR 'long-term':ti,ab
  OR '1 year':ti,ab
  OR '12 month*':ti,ab
  OR 'beyond 90 days':ti,ab
  OR '90 days to 1 year':ti,ab
  OR 'delayed recovery':ti,ab
  OR 'delayed functional improvement':ti,ab
  OR 'late functional deterioration':ti,ab
  OR 'functional trajectory':ti,ab
  OR 'modified rankin scale':ti,ab
  OR mrs:ti,ab
)
```

**14.3 Web of Science Core Collection**

```
TS=(
  (
    "stroke"
    OR "acute ischemic stroke"
    OR "ischemic stroke"
    OR "large vessel occlusion"
    OR LVO
    OR "basilar artery occlusion"
    OR BAO
    OR "vertebrobasilar occlusion"
    OR VBAO
  )
  AND
  (
    "endovascular thrombectomy"
    OR "mechanical thrombectomy"
    OR EVT
    OR "endovascular treatment"
    OR "endovascular therapy"
    OR "stent retriever"
    OR "aspiration thrombectomy"
  )
  AND
  (
    "treatment outcome"
    OR "follow-up"
    OR "long-term"
    OR "1 year"
    OR "12 month*"
    OR "beyond 90 days"
    OR "90 days to 1 year"
    OR "delayed recovery"
    OR "delayed functional improvement"
    OR "late functional deterioration"
    OR "functional trajectory"
    OR "modified Rankin Scale"
    OR mRS
  )
)
```

**14.4 Cochrane Library**

#1 ("stroke" OR "acute ischemic stroke" OR "ischemic stroke" OR "large vessel occlusion" OR LVO OR "basilar artery occlusion" OR BAO OR "vertebrobasilar occlusion" OR VBAO):ti,ab,kw

#2 ("endovascular thrombectomy" OR "mechanical thrombectomy" OR EVT OR "endovascular treatment" OR "endovascular therapy" OR "stent retriever" OR "aspiration thrombectomy"):ti,ab,kw

#3 ("long-term" OR "1 year" OR "12 month*" OR "beyond 90 days" OR "90 days to 1 year" OR "delayed recovery" OR "delayed functional improvement" OR "late functional deterioration" OR "functional trajectory" OR "modified Rankin Scale" OR mRS):ti,ab,kw

#4 MeSH descriptor: [Stroke] explode all trees
 OR MeSH descriptor: [Brain Ischemia] explode all trees
 OR MeSH descriptor: [Thrombectomy] explode all trees
 OR MeSH descriptor: [Treatment Outcome] explode all trees
 OR MeSH descriptor: [Follow-Up Studies] explode all trees

#5 #1 AND #2 AND (#3 OR #4)
