# Supplementary material for: Functional trajectories beyond 90 days after endovascular thrombectomy: a systematic review with primary synthesis and supportive narrative evidence
Source: Front Neurol. 2026 Aug 3;17:1847994. doi: 10.3389/fneur.2026.1847994 (PMC13482335; doi:10.3389/fneur.2026.1847994)
Supplement: Supplementary file 2 [file Table_1.docx]

**Supplementary Table S1. Studies retained for discussion but not included in the primary synthesis**

| **Study** | **Population / stroke type** | **Follow-up** | **Main focus** | **Reason not included in primary synthesis** | **Suggested use** |
| --- | --- | --- | --- | --- | --- |
| Xu et al., 2024 | VBAO patients treated with EVT | 90 days, 1 year | INR-related measures and outcomes | Prognostic biomarker study rather than direct within-patient transition beyond 90 days. | Adjacent supportive evidence on long-term outcome after EVT. |
| Xiao et al., 2022 | VBAO patients treated with EVT (PERSIST) | 3 months, 1 year | Renal impairment and clinical outcomes | Prognostic factor study rather than a post-90-day trajectory study. | Long-term risk stratification after EVT. |
| Li et al., 2024 | Basilar artery occlusion; ATTENTION 1-year follow-up | 90 days, 1 year | Sustained efficacy of EVT versus control at 1 year | Assessed durability of treatment benefit rather than within-patient post-90-day transition. | Long-term durability of EVT benefit. |
| Huijberts et al., 2024 | Late-window anterior circulation LVO (MR CLEAN-LATE) | 90 days, 2 years | Two-year follow-up of EVT versus best medical treatment | Long-term randomized efficacy follow-up rather than direct trajectory analysis beyond 90 days. | Persistence of EVT benefit beyond 1 year. |
| Thomalla et al., 2024 | Large-core anterior circulation stroke (TENSION) | 90 days, 12 months | Twelve-month outcome, quality of life, and survival | Provided long-term efficacy data without specifically quantifying transition beyond 90 days. | Sustained long-term benefit of EVT in large infarcts. |
| Hu et al., 2024 | VBAO patients treated with EVT | 90 days, 1 year | ASCOD phenotyping and outcomes | Etiology-focused outcome study rather than trajectory-focused study. | Etiological heterogeneity and outcome interpretation. |
| Wang et al., 2022 | VBAO patients treated with EVT | 3 months, 1 year | Initial symptom clusters and outcomes | Symptom-phenotype study, not designed to analyze post-90-day functional change. | Baseline phenotype and long-term prognosis. |
| Wu et al., 2021 | VBAO patients receiving EVT | 3 months, 1 year | Predictors of 3-month and 1-year mortality | Mortality-prediction study rather than post-90-day functional trajectory study. | Long-term mortality predictors after EVT. |
| Park et al., 2025 | Anterior circulation ICAS-related LVO with rescue stenting | 3 months, >=12 months | Long-term outcome of rescue stenting after failed MT | Technique-specific rescue therapy study rather than natural recovery trajectory study. | Durability of rescue endovascular strategies. |
| Gao et al., 2025 | LVO with rescue balloon angioplasty/stenting | 90 days, 1 year | One-year outcomes of rescue treatment strategy | Treatment-strategy comparison study rather than post-90-day trajectory study. | Long-term impact of rescue therapy. |
| Geng et al., 2025 | Young versus older VBAO patients after EVT | 90 days, 1 year | Age-stratified long- term outcomes | Subgroup comparison study rather than direct late-trajectory study. | Age-related heterogeneity in long- term outcome. |

*Note. Studies in Supplementary Table S1 were retained because they were relevant to the broader topic of long-term outcome after EVT but did not directly assess within-patient functional transition beyond the conventional 90-day endpoint.*
