# Supplementary material for: Functional trajectories beyond 90 days after endovascular thrombectomy: a systematic review with primary synthesis and supportive narrative evidence
Source: Front Neurol. 2026 Aug 3;17:1847994. doi: 10.3389/fneur.2026.1847994 (PMC13482335; doi:10.3389/fneur.2026.1847994)
Supplement: Supplementary file 3 [file Table_2.docx]

**Supplementary Table S2. Full-text reports excluded, with reasons**

*Note.* Full-text reports were excluded if they did not directly evaluate within-patient functional trajectories beyond 90 days after EVT, focused primarily on prognostic factors, treatment durability, rescue strategies, non-EVT populations, or represented reviews, protocols, guidelines, or health economic analyses.

| **No.** | **First author** | **Year** | **Short title / study focus** | **Main reason for exclusion** |
| --- | --- | --- | --- | --- |
| 1 | Equiza | 2022 | Adaptive stroke unit–based system of care | Not a direct post-90-day functional trajectory study |
| 2 | von Danwitz | 2025 | Delayed functional independence and dependence after thrombectomy | Assessed discharge-to-90-day transition rather than post-90-day trajectory |
| 3 | Mortezaei | 2025 | Long-term (12 months) outcomes of EVT for large stroke | Review / meta-analysis rather than original study |
| 4 | Song | 2023 | Locked-in syndrome responding to EVT | Special population study; not a direct post-90-day trajectory study |
| 5 | Nagaratnam | 2024 | e-Stroke AI decision support software | Workflow / pathway study; no direct post-90-day trajectory assessment |
| 6 | Ramazanoglu | 2025 | Tandem lesions with emergent stenting | Rescue strategy / technique-specific study |
| 7 | Qian | 2025 | Biological age acceleration after EVT | Prognostic factor study only |
| 8 | Tan | 2026 | ABAO with NIHSS ≤10: EVT versus SMT | Treatment-effect durability / long-term efficacy study only |
| 9 | Huo | 2024 | Sex differences in outcomes of EVT for VBAO | Not a direct post-90-day functional trajectory study |
| 10 | Chen | 2022 | 24-h NIHSS predicts outcomes after EVT | Prognostic factor study only |
| 11 | Gu | 2022 | Perioperative glycemic indicators in VBAO EVT | Prognostic factor study only |
| 12 | Han | 2024 | Cost-effectiveness of EVT in China | Review / protocol / guideline / health economic study |
| 13 | Huang | 2022 | IV thrombolysis plus stent thrombectomy | Other reasons (short follow-up / not aligned with target design) |
| 14 | Jeong | 2013 | Stent-assisted thrombectomy versus AMCD | Rescue strategy / technique-specific study |
| 15 | Kim | 2022 | Annual EVT case volume and 1-year mortality | Not a direct post-90-day functional trajectory study |
| 16 | Li | 2024 | 3-month DAPT after MT | Rescue strategy / technique-specific study |
| 17 | Li | 2025 | Posterior circulation tandem lesions and stenting | Rescue strategy / technique-specific study |
| 18 | Luo | 2022 | Rescue treatment after failed MT in BAO | Rescue strategy / technique-specific study |
| 19 | Murphy | 2026 | Inter-hospital transfer before EVT | Not a direct post-90-day functional trajectory study |
| 20 | Peng | 2024 | Stress hyperglycemia ratio in ABAO EVT | Prognostic factor study only |
| 21 | Combeau | 2025 | CIA on target vessel treated with MT | Not a direct post-90-day functional trajectory study |
| 22 | Sahakyan | 2023 | Stroke service implementation in Armenia | Wrong population / not EVT-specific |
| 23 | Pilato | 2020 | Functional outcome in NICU stroke after IAMT | Not a direct post-90-day functional trajectory study |
| 24 | Shi | 2014 | Functional dependence despite successful revascularization | Not a direct post-90-day functional trajectory study |
| 25 | Staszewski | 2026 | Cerebrolysin after EVT 12-month outcomes | Treatment-effect durability / long-term efficacy study only |
| 26 | Wang | 2024 | Metabolic syndrome in VBAO EVT | Prognostic factor study only |
| 27 | Yang | 2024 | Stress hyperglycemia after MT mortality | Prognostic factor study only |
| 28 | Zaidat | 2023 | TESLA protocol | Review / protocol / guideline / health economic study |
| 29 | Aoki | 2016 | Long-term outcome after tPA | Wrong population / not EVT-specific |
| 30 | Achit | 2017 | Cost-effectiveness THRACE | Review / protocol / guideline / health economic study |
| 31 | Verschoof | 2022 | Active cancer after EVT | Not a direct post-90-day functional trajectory study |
| 32 | Mechtouff | 2022 | sST2 after MT | Prognostic factor study only |
| 33 | Garg | 2023 | Maternal stroke recurrence in pregnancy | Wrong population / not EVT-specific |
| 34 | Morsi | 2024 | EVT with or without IVT cost-effectiveness | Review / protocol / guideline / health economic study |
| 35 | An | 2024 | Remote ischemic conditioning in sICAS | Wrong population / not EVT-specific |
| 36 | Wang | 2024 | Moderate stroke antiplatelet study | Wrong population / not EVT-specific |
| 37 | Schirmer | 2023 | Antiplatelets and antithrombotics guideline update | Review / protocol / guideline / health economic study |
| 38 | Chen | 2017 | Subacute recanalization case series | Wrong population / not EVT-specific |

*Abbreviations:* EVT, endovascular thrombectomy; VBAO, vertebrobasilar artery occlusion; BAO, basilar artery occlusion; ABAO, acute basilar artery occlusion; MT, mechanical thrombectomy; SMT, standard medical treatment; DAPT, dual antiplatelet therapy.
